# Development of a non-specialist worker delivered psychological intervention to address alcohol use disorders and psychological distress among conflict-affected populations in Uganda and Ukraine

**DOI:** 10.1186/s13033-024-00656-4

**Published:** 2025-01-08

**Authors:** Abhijit Nadkarni, Alessandro Massazza, Wietse A. Tol, Sergiy Bogdanov, Lena S. Andersen, Quincy Moore, Bayard Roberts, Helen A. Weiss, Soumya Singh, Melissa Neuman, Carl May, Daniela C. Fuhr

**Affiliations:** 1 Centre for Global Mental Health, Department of Population Health, London School of Hygiene and Tropical Medicine, London, UK WC1E 7HT, UK; 2 Department of Health Services Research and Policy, London School of Hygiene and Tropical Medicine, London, UK; 3 Department of Public Health, University of Copenhagen, Copenhagen, Denmark; 4 Centre for Mental Health and Psychosocial Support, National University of Kyiv-Mohyla Academy, Kyiv, Ukraine; 5 Department of Infectious Disease Epidemiology and International Health, London School of Hygiene and Tropical Medicine, London, UK; 6 Addictions and Related Research Group, Sangath, India; 7 Department of Prevention and Evaluation, Leibniz Institute for Prevention Research and Epidemiology, Bremen, Germany; 8 United for Global Mental Health, Somerset House, Strand, London, WC2R 1LA UK

**Keywords:** Alcohol use disorder, Non-specialist worker, Task sharing, Uganda, Ukraine, Conflict setting, Psychological treatment

## Abstract

**Background:**

Despite the significant burden of alcohol use disorders (AUD), there is a large treatment gap, especially in settings and populations affected by armed conflict. A key barrier to care is the lack of contextually relevant interventions and adequately skilled human resources to deliver them. This paper describes the systematic development of the CHANGE intervention, a potentially scalable psychological intervention for people with co-existing AUD and psychological distress in conflict-affected populations, delivered by non-specialist workers (NSWs).

**Methods:**

CHANGE was developed in sequential steps: (1) identifying potential treatment strategies through a meta-review and Delphi survey with international experts; (2) in-depth interviews (IDIs) with key stakeholders from the study settings in Uganda and Ukraine; and (3) three consultative workshops with international experts and experts from Uganda and Ukraine to develop a theoretical framework for the intervention informed by outputs of the Delphi and IDIs.

**Results:**

In the Delphi survey, experts reached agreement on the acceptability, feasibility and potential effectiveness of the following components: identify high-risk situations, problem solving skills, assessment, handling drinking urges, communication skills, pros and cons of drinking, and identifying high-risk situations. From the IDIs we identified (a) causal attributions for using alcohol e.g., psychosocial stressors; (b) cultural norms related to alcohol consumption such as patriarchal stereotypes; and (c) coping strategies to deal with drinking problems such as distraction. The CHANGE intervention developed through the consultative workshops can be delivered in three sequential phases focussed on assessment, feedback, and information (Phase 1); providing the client with need-based skills for dealing with high-risk situations related to alcohol use (Phase 2), and relapse prevention and management (Phase 3).

**Conclusions:**

CHANGE is a contextually relevant and potentially scalable treatment for co-existing AUD and psychological distress to be delivered by NSWs to conflict-affected populations. Effectiveness and cost-effectiveness of CHANGE will be tested in Uganda and Ukraine.

**Supplementary Information:**

The online version contains supplementary material available at 10.1186/s13033-024-00656-4.

## Introduction

The United Nations High Commissioner for Refugees estimated that 80 million people were forcibly displaced due to conflict and violence, or as a result of disasters as of December 2019 [1]. These displaced individuals are disproportionately affected by adversities such as acute food insecurity and malnutrition, poor housing with basic living conditions, and limited access to services and employment [1]. These significant pre- and post-migration stressors during their flight to safety increase vulnerability to mental health problems [2]. Approximately one in five people in conflict-affected settings experience depression, anxiety disorders, post-traumatic stress disorder (PTSD), bipolar disorder, or schizophrenia [3]. This is much higher than the mean global prevalence of one in 14 [4]. There are less data on alcohol problems among conflict-affected populations but evidence shows that among internally displaced persons and refugees, the prevalence of hazardous/harmful alcohol use may range from 17 to 36% [5]. As many as one in three forced migrants may be using alcohol in harmful or hazardous ways, and, when measured among current drinkers only, this estimate can be as high as two in three drinkers [5].

While the terms alcohol-related problems and alcohol use disorders (AUDs) are often used interchangeably, they refer to different concepts in the context of alcohol consumption and its impact on an individual’s life. The former refer to a wide range of issues that arise due to alcohol use but do not necessarily indicate a formal diagnosis of a disorder; and may involve situations such as accidents or injuries, health issues stemming from excessive drinking, and disruptions in social or family relationships. On the other hand, AUD is a formal medical diagnosis characterised by a pattern of alcohol consumption that drinking more or for longer than intended, unsuccessful efforts to cut down or control drinking, spending a lot of time drinking or recovering from alcohol use, and experiencing withdrawal symptoms when not drinking. Mental disorders are closely linked with alcohol use disorders (AUD) - psychological distress increases risk of AUD, and vice versa; and there are common pathophysiological pathways and risk factors leading to the development and impact of psychological distress and AUD [6]. There is a substantial evidence base demonstrating the association between AUD and common mental disorders such as anxiety, depression and PTSD [6–8], and this is true for conflict-affected populations as well [9]. These disorders might result from direct exposure to violent and traumatic events, as well as from ongoing daily stressors in their new areas of settlement such as impoverishment, unemployment, poor living conditions, social isolation and discrimination [10, 11].

Mental health and psychosocial support (MHPSS) programmes, defined as ‘any type of local or outside support that aims to protect or promote psychosocial well-being and/or prevent or treat mental disorder’ [12], are a common component of humanitarian response [13]. However, a critical gap in such programmes is availability of services for people with AUDs due to resource limitations, provider knowledge and capacity, instability, disruptions to health systems, and attrition of healthcare workers [14]. Another key challenge is lack of available evidence-based and scalable interventions for AUDs in conflict-affected settings [15]. This is partly because existing AUD interventions are for general populations and not considered appropriate for conflict-affected populations as they are not adapted to their particular sociocultural context, do not address other forms of psychosocial distress, and rely on mental health professionals of whom there are very few in such settings. Consequently, while the MHPSS guidelines for humanitarian settings, such as the Inter-Agency Standing Committee guidelines [12], recommend action to address AUD in conflict-affected settings they provide very little detail; and AUD interventions are typically not included in humanitarian responses [14].

Problem Management Plus (PM+) is a low-intensity intervention designed to reduce psychological distress among people exposed to adversity [16]. It is a trans-diagnostic intervention to be delivered by non-specialist providers over five sessions and targets symptoms of anxiety and depression, using strategies such as problem-solving, stress management, behavioural activation, and social support. PM + has been demonstrated to be effective [17–19], and adopted by the WHO as the key potentially scalable psychological intervention to be implemented and scaled-up within primary and community health care settings [20]. However, PM + does not contain psychological strategies that directly address AUD, which commonly co-occur with anxiety and depression, especially among conflict-affected populations.

The work described in this paper is a part of the Al**C**ohol use in **H**umanitari**AN** settings: a programme of work to address alcohol use disorders and associated adversities among conflict-affected populations in U**G**anda and Ukrain**E** (CHANGE) [21]. The CHANGE programme seeks to develop and implement a psychological intervention that can address the needs of people with co-existing psychological distress and AUD. The emphasis is on developing an intervention that is both acceptable to patients and feasible for delivery by non-specialist workers (NSWs) in conflict-affected settings. The aim of this paper is to describe the systematic process used to develop the CHANGE intervention.

## Methods and results

**Fig. 1:**
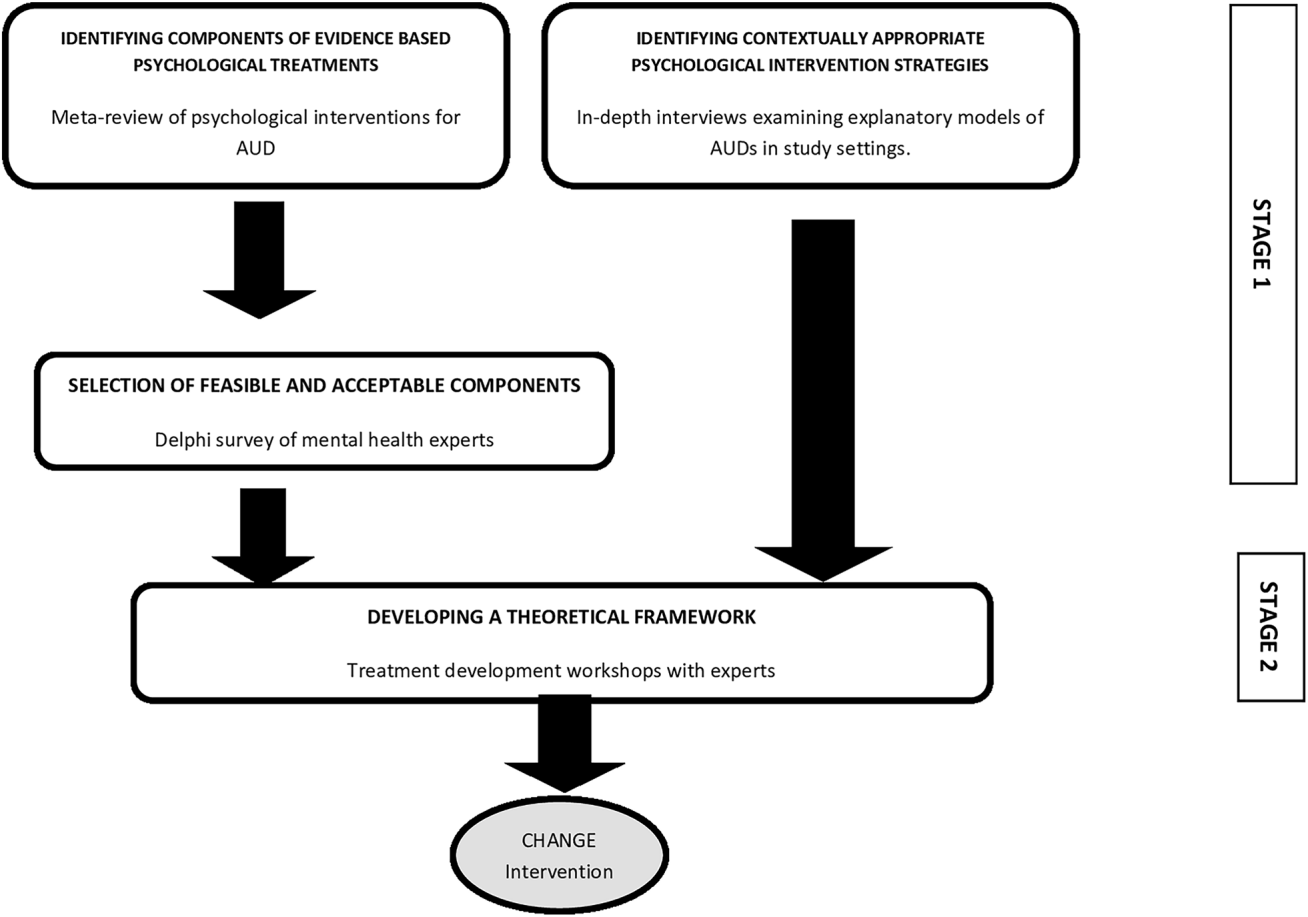
An overview of the intervention development process

**Fig. 2:**
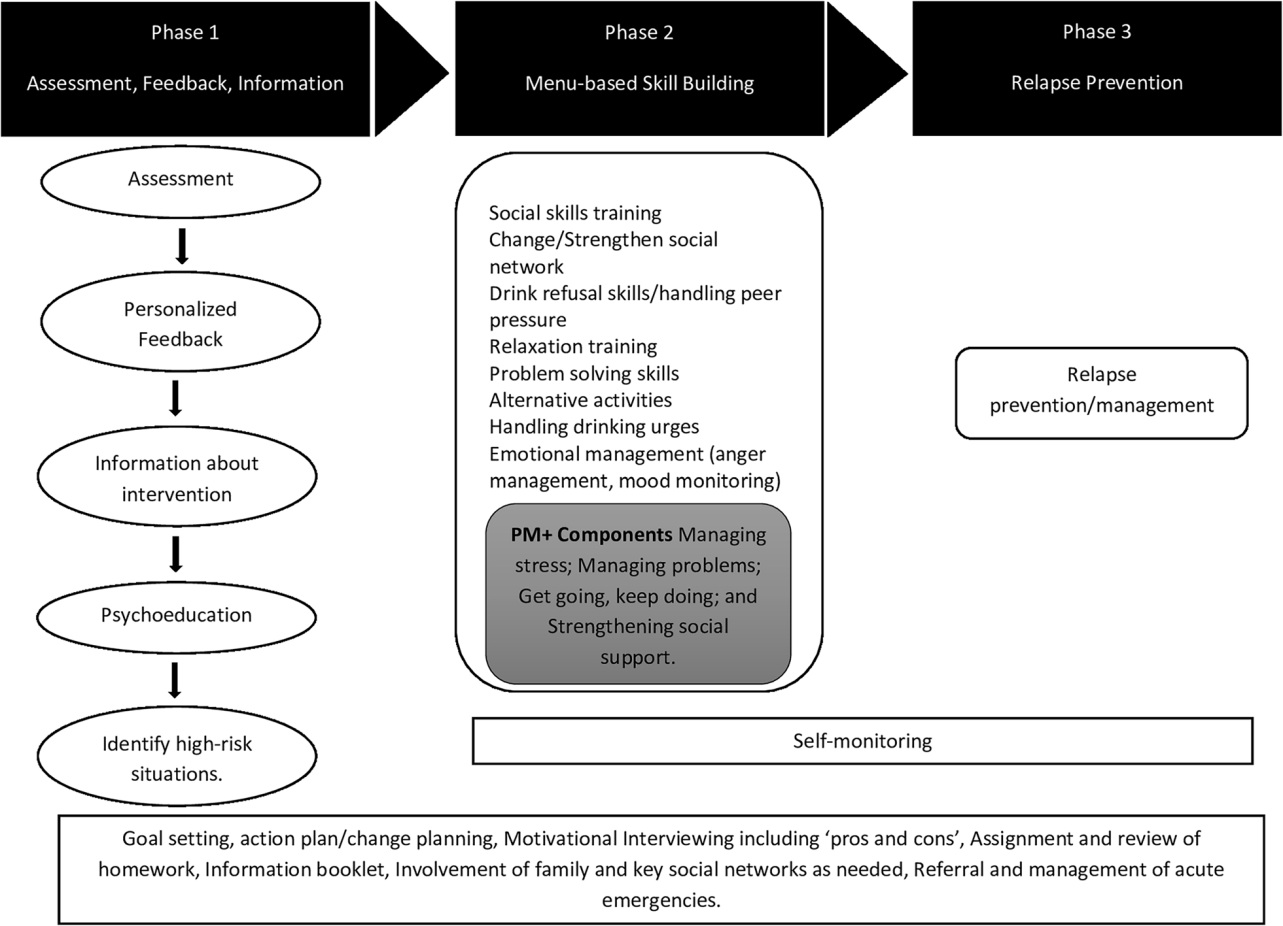
Conceptual framework of the CHANGE intervention

The intervention development process was informed by the Medical Research Council (MRC) UK framework for developing and evaluating complex interventions [22], the approach of ‘dismantling’ existing evidence-based psychological treatments to ‘distil’ individual treatment components suitable for task-sharing [23], and the methodology adopted by colleagues in India for the development of psychological interventions for delivery by lay counsellors [24]. Our methodology, illustrated in Fig. 1, involved two sequential stages, with distinct procedures within each stage. Broadly the stages involved: (1) identification of potential psychological intervention components; and (2) development of a theoretical framework underpinning the new intervention. In the following sections we describe the methods and outputs at each stage of intervention development culminating in the conceptual framework of the final intervention (Fig. 2).

### Stage 1: Identifying potential psychological intervention components

The aim of this first step was to identify AUD intervention components which would be potentially suitable as components for CHANGE with regard to their effectiveness, evidence of their acceptability to the target population, and feasibility of delivery by NSWs.

#### Identifying common components of empirically supported psychological interventions

We conducted a review of systematic reviews (meta-review) to identify common components from evidence-based psychological interventions for AUDs. We identified relevant primary studies from eligible systematic reviews and extracted information about the components from the interventions from these studies. Details of this meta-review have been published elsewhere [25]. In brief, we identified eight reviews which demonstrated the effectiveness of the following psychological interventions: behavioural couples therapy, cognitive behaviour therapy (CBT) combined with motivational interviewing (MI), brief interventions (BI), contingency management, psychotherapy plus BI, Alcoholics Anonymous and 12-Step Treatment programmes (TSP), family-therapy or family-involved treatment, and community reinforcement approach (CRA). For our intervention development, we were not interested in the evidence-based treatment *classes* (composites of individual strategies e.g., CBT*)* but their component *strategies* (e.g., cognitive restructuring) as described in the source paper. We synthesised the components further by merging similar ones and removed those which reflected *techniques* (specific procedures used to implement a strategy e.g., self-monitoring).

Table 1 summarises the components that we extracted from the evidence-based psychological treatments. The most used components in effective interventions for AUDs (included in *≥* 10 trials) were assessment, personalised feedback, MI, goal setting, setting and review of homework, problem solving skills, and relapse prevention/management.

**Table 1:** Components from evidence-based psychological interventions for AUDs [25]

Reported in *≥* 10 trials	Reported in 5–9 trials	Reported in 2–4 trials	Reported in only one trial
• Assessment • Personalized feedback • Motivational interviewing • Goal setting • Setting and review of homework • Problem solving skills • Relapse prevention/management	• Communication skills • Self-monitoring • Changing social networks • Emotional management • Handling drinking urges • Pros and cons of drinking • Action planning/change plan • Assertiveness training • Alternative activities • Identifying high risk situations • Coping skills to deal with high-risk situations • Drink refusal skills/handling peer pressure • Contingency management • Information booklet	• Relationship enhancement exercise • Psychoeducation • Direct advice on reducing drinking • Daily mood monitoring • Cognitive restructuring • Social skills training • Relaxation training • Abstinence contracting • Anger management	• Decision making skills • Dealing with accusation of relapse • Enhancing self-esteem • Stress management • Behavioral self-control training • Importance/confidence • Identifying and disputing distorted thoughts (Cognitive restructuring) • Cognitive self-management for reducing negative thoughts

#### Selection of feasible, acceptable, and potentially effective components

We conducted a Delphi survey to identify intervention components which would be most feasible for delivery by NSWs, would be acceptable to people with AUD in conflict-affected settings, and perceived to be potentially effective when delivered by NSWs. The survey was conducted with individuals having expertise in research of and/or clinical use of psychological interventions for AUD. We used a digital survey platform on which the participants rated each of the thirty components reported in two or more trials (Table 1) on a five-point Likert scale (1 = Least to 5 = Most) on three dimensions: *Feasibility* for delivery by adequately trained and supervised NSWs; *Acceptability* to people with AUD in conflict settings; and perceived *Effectiveness* in bringing about an observable change in AUD.

We purposively selected and invited 43 experts in the field of AUD intervention delivery and/or research. Of these, 25 (58.1%) from 11 countries (Australia, Brazil, Georgia, Germany, India, Mexico, South Africa, Tanzania, Ukraine, UK, USA) responded to Round 1 of the survey. This included 40% men and 60% women, with mean age 46 years, an average of 17 years of research experience, and an average of 18 years of mental health service provision experience. In Round 1, agreement (defined as all participants rating 4 or 5) was reached for the following components: feasibility only (Assessment, Psychoeducation); acceptability only (Motivational interviewing, Relapse prevention management); feasibility and acceptability (Coping skills to deal with high-risk situations); and perceived effectiveness (Handling drinking urges). In Round 2, the experts were presented with responses from other participants (as histograms) and asked to re-rate all components, except for those components on dimensions for which agreement was reached in Round 1. In Round 2, we received responses from 21 (84%) out of 25 participants. In Round 2, the experts reached agreement on the following components: feasibility only (Identify high-risk situations, problem solving skills); acceptability only (Assessment, handling drinking urges); feasibility and acceptability (Communication skills, pros and cons of drinking); acceptability and effectiveness (Identifying high-risk situations). Table 2 summarizes all the components on which the experts reached agreement after Round 2.

**Table 2:** Expert agreement on feasibility, acceptability, and perceived effectiveness

Component	Feasible	Acceptable	Effective
Problem solving skills	✓		
Psychoeducation	✓		
Motivational interviewing		✓	
Relapse management		✓	
Assessment	✓	✓	
Communication skills	✓	✓	
Coping skills to deal with high-risk situations	✓	✓	
Pros and cons of drinking	✓	✓	
Handling drinking urges		✓	✓
Identify high-risk situations	✓	✓	✓

#### Mapping contextually appropriate psychological intervention components

To define the contextually relevant content of the intervention, we aimed to: (a) examine the reasons for drinking within the study settings; (b) understand the cultural/contextual norms that facilitate and inhibit drinking; (c) describe the adverse consequences of the drinking; and (d) explore the coping strategies used by individuals to manage their heavy drinking. This was done using in-depth interviews (IDIs) in Uganda and Ukraine.

In Ukraine, participants were recruited from the urban cities of Kyiv and Dnipro and smaller towns throughout eastern and western Ukraine. In Uganda, participants were recruited from the Rhino Camp refugee settlement located in north-western Uganda in the West Nile districts of Terego and Madi Okollo. We selected the two country sites because of the following reasons: (a) our previous work in these settings identified high levels of alcohol problems, (b) stakeholders in those settings had expressed a strong need for addressing drinking problems, (c) the two settings enabled us to focus on two different types of conflict-affected populations (i.e., refugees and internally displaced populations), and (d) for the WHO to recommend scale-up of an intervention it needs to have demonstrated evidence of effectiveness through trials in two distinct settings.

The project team worked with a network of local community organizations, and service providers. Participants were recruited via purposive and snowball sampling, as well as through referrals from community workers and organizations. A total of 66 participants were interviewed in Ukraine. This included 25 conflict-affected men who experienced problematic drinking (10 internally displaced persons, five persons living in an area that experienced active military action, and 10 veterans), family members of conflict-affected men who experienced problematic drinking (*n* = 15), and healthcare providers who provided care for conflict-affected men with problematic drinking (*n* = 26). A total of 57 participants were interviewed in Uganda. This included male refugees with problematic drinking (*n* = 17), family members of men with problematic drinking (*n* = 15), refugee leaders and religious leaders (e.g., block leaders, refugee welfare council leaders, and leaders of various religious denominations in the settlements) (*n* = 15), and MHPSS service providers in the refugee settlement (e.g., those working in the government primary health care facilities and non-governmental organisations (NGOs) operating in the settlement) (*n* = 10).

The interview guides (Appendix A) were developed to assess perceptions of alcohol use, services available for alcohol problems, and help-seeking behaviour. Interviews were conducted by experienced qualitative interviewers who were fluent in Ukrainian and Russian (Ukraine study), and Juba Arabic and English (Uganda study). Data were collected until saturation was reached (i.e., until new themes were not emerging from the data) The detailed findings of the IDIs are being written-up as a separate publication and the key findings from preliminary thematic analysis as triangulated from the two sites are summarised here. The IDIs were transcribed, and a coding framework was developed using a subset of the transcripts. The entire dataset was then coded using the coding framework and ongoing discussions took place with other investigators to discuss the analysis and interpretation of the data.

The broad *causal attributions* for using alcohol were: (1) Psychosocial stressors (e.g., Difficulties with psychological adaptation to a new situation, demoralisation, loss of social contacts, highly uncertain future, poor living conditions), (2) specific mental disorders such as PTSD and depression, (3) socio-economic insecurity, (4) difficulties in coping with stress and to relax, (5) stigmatization of veterans (in Ukraine), (6) peer pressure, (7) ease of access (e.g. alcohol available on credit), (8) supernatural causes (e.g., witchcraft), (9) the hereditary nature of AUDs, and (10) complex reasons (multiple reasons). Some causal attributions specific to conflict-affected men from Ukraine included: (1) Continuation of drinking habits picked up in the army; (2) Perceived benefits of alcohol such as alcohol as medication to reduce physical pain and aid sleep, as a replacement for the adrenalin rush, for enjoyment, and for feeling empowered.

*Cultural norms* related to alcohol consumption included: (1) Alcohol problems not being recognized in the community; (2) Taboo around public discussion of alcohol use; (3) Alcohol problems as socially appropriate with a strong social/cultural influence and involving social reciprocity behaviors; (4) Normalization of the availability of alcohol; (5) Easily available self-crafted alcohol; (6) Association of alcohol with patriarchal stereotypes (e.g. masculinity, strength); and (7) Traditions encouraging the use of alcohol such as alcohol being viewed as part of everyday life in military service (Ukraine only), part of social activities and events, needed for relaxation, refusal of drinks as a sign of disrespect, transactional use of alcohol, and tolerance of heavy alcohol use in public.

*Impact* of AUD was experienced through family neglect, consequences for physical (e.g. seizures) and mental (e.g., multiple addictions, depression, aggressiveness, self-harm/suicide) health, loss of life, difficulties with job, violence (particularly intimate partner violence), financial difficulties, loss of support (family doctor refusing to treat, family conflict, social isolation), loss of dignity or standing in the community, and stigma.

*Coping strategies* to deal with their drinking problems included self-awareness and self-control, decision not to drink, distraction (e.g., pets, household activities, sports), seeking a supportive environment, taking medications to stop drinking, finding alternatives to relieve stress, and drinking non-alcoholic beverages.

Table 3 maps the identified intervention components as responses to the contextual findings from the IDIs.

**Table 3:** Identified intervention components mapped against contextual findings from the in-depth interviews (IDIs)

Findings from IDIs	Intervention components identified in meta review
*Reasons for drinking *	
Difficulties with psychological adaptation to new situation	Problem solving, stress management
Difficulties raising a child	Problem solving
Family conflict	Social skills training
Feeling alone because of loss of social contacts	Strengthening social networks
Socio-economic insecurity	Problem solving
Difficulties to cope with/reduce stress	Stress management
Peer pressure	Drink refusal skills
Ease of access to alcohol	Handling drinking urges
Witchcraft	Psychoeducation
Hereditary reasons	Psychoeducation
Complex reasons (multiple reasons)	Various components
Experience of drinking alcohol in the army*	Alternative activities
Perceived benefit of alcohol - Alcohol as medicine*	Psychoeducation
Perceived benefit of alcohol - Replacement for adrenalin rush*	Psychoeducation
Perceived benefit of alcohol - Enjoyment*	Alternative activities
Perceived benefits of alcohol - Feeling empowered*	Psychoeducation
*Impact*	
Family neglect	Relation enhancement exercises
Consequence for physical health	Psychoeducation
Loss of life	Psychoeducation
Consequences for mental health	Psychoeducation
Difficulties with job	Psychoeducation
Violence	Anger management
Financial difficulties	Problem solving
Loss of support	Strengthening social networks
Loss of dignity or standing in the community, stigma	Psychoeducation
*Coping strategies*	
Self-awareness	Self-monitoring
Decides not to drink	Motivational interviewing (MI)
Has something else to do instead of drinking	Alternative activities
Seek supportive environment	Strengthening social networks
Takes medications for alcohol use disorders (AUD)	Referral
Uses alternatives to alcohol to relieve stress	Stress management
Drinking other beverages	Handling drinking urges
Self-control	MI

*Only in conflict-affected men from Ukraine

There are certain findings from the IDIs that are not listed in Table 3 as they were beyond the scope of an individual level intervention (e.g., highly uncertain future, living in conditions of war, poor living conditions, stigmatisation of veterans). These broader structural issues are best addressed at programmatic level through policy and community interventions such as improved protection for refugees, strengthening access to basic needs, and awareness building around stigma. These include factors such as social acceptability of alcohol problems due to social/cultural influence, social reciprocity and easy availability of alcohol, patriarchal stereotypes around drinking, traditions encouraging the use of alcohol, and taboo around discussion about alcohol.

### Stage 2: developing a theoretical treatment framework

The goal of this stage was to develop a theoretical framework for the CHANGE intervention based on the intervention components identified in the meta-review. This involved three treatment development workshops in September 2021 where Ugandan and Ukrainian mental health experts and international experts assembled the intervention components into a coherent treatment format. The workshops were conducted virtually over Zoom.

In addition to the program investigators, five participants from Uganda, five from Ukraine and 12 international experts (from Australia, Germany, India, Kenya, Mexico, and UK; and WHO representatives) participated in the workshops. These included a mix of psychologists and psychiatrists with extensive experience of working with individuals with AUD and/or mental health problems, with some having experience of working with conflict-affected populations.

The participants were first presented with outputs of the meta-review, IDIs and Delphi survey. They were then presented with the components identified in the meta-review and asked to finalize the list of components, to organize them into related groups, and schedule them into a coherent treatment delivery framework. They had to consider their own experience/expertise, the findings from the IDIs and the Delphi survey while making the decisions through facilitated discussion. Participants could exclude components or merge components after giving a rationale for doing so. The Padlet online software (https://padlet.com/) was used to facilitate the discussion through visual maps and arrive at the conceptual framework of the intervention. Table 4 lists the components that were excluded through consensus in at least two of the three workshops.

**Table 4:** Intervention components excluded in at least two workshops

Excluded components	Workshop 1	Workshop 2	Workshop 3	Rationale
Abstinence contracting	X	X	X	Controlled drinking is a valid goal as well; abstinence as the only goal will not be consistent with principles of Motivational Interviewing
Cognitive restructuring	X	X	X	Too long for a brief therapy; too complex for non-specialist workers (NSWs)
Contingency management	X	X	X	Difficult to implement in emergency settings; expensive; not sustainable
Daily mood monitoring	X	X	X	Difficult to implement
Anger management	X		X	Included in emotional management; Concerns that it would take too much time
Assertiveness training		X	X	Too long for a brief therapy; only drink refusal skills can be included in the therapy
Communication skills		X	X	Too long for the brief therapy; too complicated for NSWs
Direct advice	X		X	Suitable for a brief intervention but not for therapy; would be inconsistent with PM + which has very clear guidance about not giving advice; important to give people options
Relationship enhancement exercises	X		X	Too long for a brief therapy

The conceptual frameworks of the intervention that were independently developed in the three workshops were then merged by the program team after qualitatively comparing and contrasting with each other (Fig. 2). The resulting CHANGE intervention is designed to be delivered in three sequential phases with distinct goals. Phase 1 is focussed on ‘Assessment, Feedback, and Information’. Accordingly, this involves detailed assessment of the drinking and its impact followed by personalised feedback, sharing of information about the intervention, psychoeducation, and identification of high-risk situations for drinking. Phase 2 aims to provide the person with need-based skills for dealing with high-risk situations related to alcohol use. These include social skills training, changing or strengthening social networks, drink refusal skills or handling peer pressure, relaxation training, problem solving skills, identifying alternative activities that do not involve drinking, handling drinking urges, and emotional management including anger management and mood monitoring. Phase 3 is centred around relapse prevention and management using the skills acquired in Phase 2. In addition, self-monitoring would be used across Phases 2 and 3 as the person works towards achieving their goal of controlled drinking or abstinence. Finally, the following components would be deployed across the whole treatment journey: MI including ‘pros and cons’, assignment and review of homework, information booklet, involvement of family and key social networks as needed, and referral and management of acute emergencies. While goal setting, and action plan/change planning would happen in Phase 1, it would need to be reviewed and iteratively revised as needed throughout the course of the intervention delivery. These components were then merged with the PM + manual while adhering to the principles of the conceptual framework described above. Some of the existing components in PM+ (Managing stress; Managing problems; Get going, keep doing [i.e., behavioural activation]; and Strengthening social support) directly configured on to some of the components identified for CHANGE and hence were modified to address AUD along with psychological distress (the original target condition for PM+).

## Discussion

This paper describes the process of development of the CHANGE intervention. CHANGE addresses co-existing psychological distress and harmful drinking among populations exposed to conflict and is designed to be delivered by NSWs. We describe the intervention development process, which started with mapping the global evidence base for effectiveness of psychological interventions for AUD. The evidence-based psychological interventions were then dismantled into their components and assessed by experts for contextual acceptability, feasibility and effectiveness when delivered by NSWs. This was supplemented with contextual evidence related to explanatory models and coping strategies used by people with AUD. Informed by both the effectiveness and contextual evidence the various components were then assembled into the CHANGE intervention by psychological treatment experts, both global and from the contexts for which the intervention is designed.

CHANGE has been informed by the PM + intervention in which the various other AUD related intervention components have been integrated. The core strategies of PM + include ‘managing stress’ (optimizing initial mastery of stress and anxiety symptoms as well as enhancing relaxation), ‘managing problems’ (taking control of problems by investing solely in problems that are of significance to one’s lives), behavioural activation (gradually re-engaging with pleasant and task-oriented activities to improve mood and functionality), strengthening social support (optimizing the capacity to re-engage in the community, eliciting support from others, and supporting oneself) [26]. In addition to these strategies delivered for psychological distress, the AUD-specific strategies in CHANGE are expected to lead to change in the following manner. The detailed assessment, personalised feedback, and psychoeducation is leveraged to facilitate a commitment to change through goal setting and change planning. The latter outlines what change they would like to make to their drinking and the actions that will have to be taken to achieve that goal, including identification of risky situations that will interfere with achievement of the goal. Subsequently the NSW and person with AUD will work collaboratively to develop the latter’s skills (e.g., drink refusal, problem solving) to make the changes they desire. Finally, they learn how to manage potential or actual relapses using these same skills.

The CHANGE intervention allows for flexibility in the number of sessions based on the diversity of patient needs, but the expectation is that it would be delivered in four to six sessions which is consistent with the requirements of low intensity psychological interventions [27]. Similarly, CHANGE allows for flexibility on the number of individual sessions utilised to achieve the goals in a particular phase. For example, in individuals with AUD who are ready for change, the movement from the first phase to the next maybe faster and could happen even in one session, while for those who are ambivalent about their drinking and drinking goals, two or more sessions might be required to navigate the first phase. While the contextual realities would be expected to define the frequency of the sessions, it is recommended that they are conducted on a weekly or fortnightly basis.

**Fig. 3:**
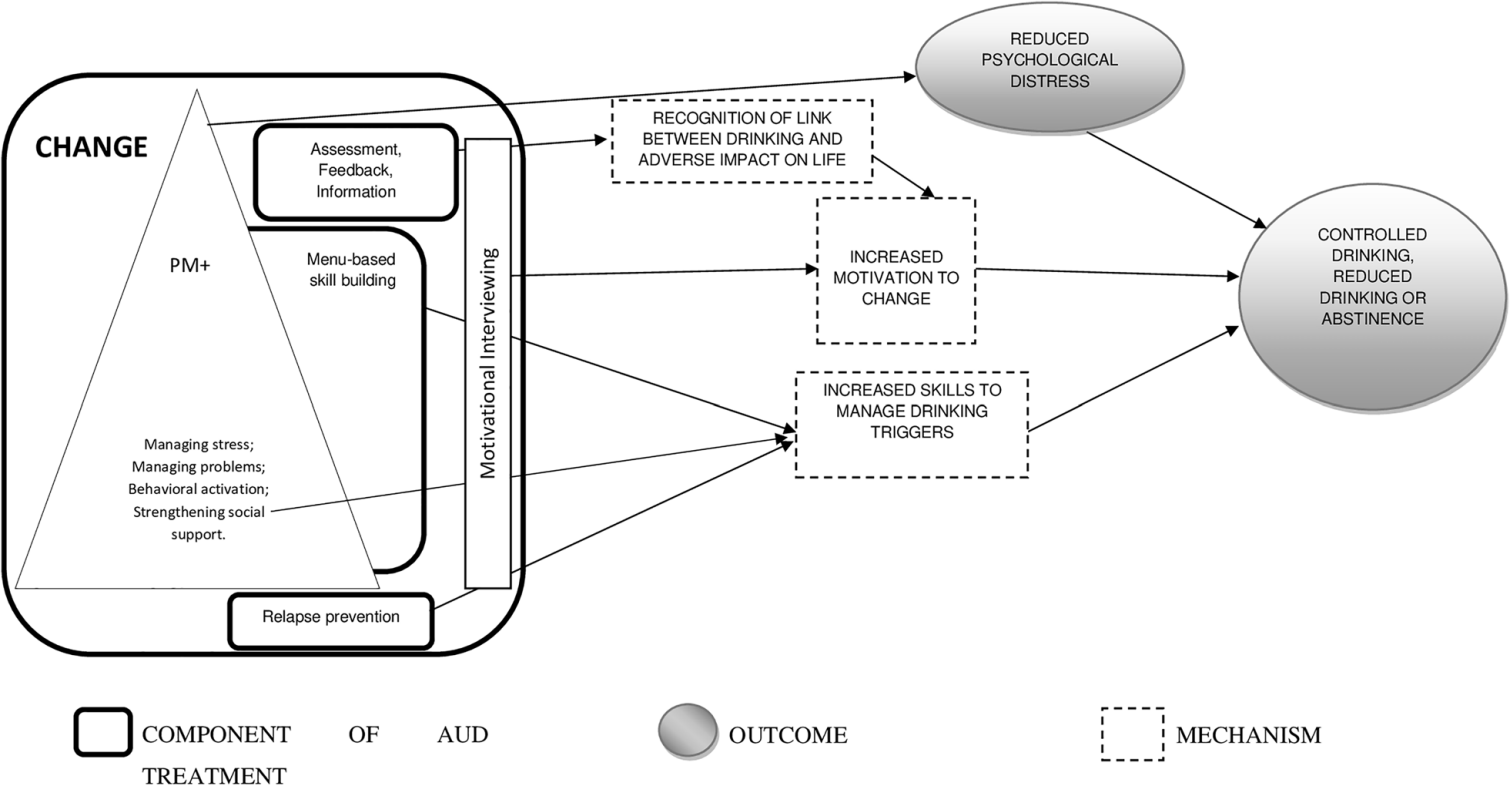
Modelling CHANGE pathways to impact

Figure 3 outlines how the CHANGE intervention is postulated to lead to change in drinking behaviours. The detailed assessment and personalised feedback are expected to increase the motivation to change drinking behaviour by facilitating the recognition of the link between drinking and the consequent adverse impacts. The various skills (including some relevant ones from PM+) will increase the capacity to manage drinking triggers, help achieve the drinking goals, and prevent relapse. Additionally, the reduction of psychological distress resulting from the original PM + components will also help in achieving the drinking goals as the former is an important trigger for initiation and continuation of drinking.

Our intervention development process has some limitations as outlined here. The review conducted as a part of the treatment development process only included papers which were published in English language journals. However, this may not be a significant limitation as most research about AUDs, including that from LMICs, is published in peer-reviewed public health and social sciences journals in English. One criticism of an intervention development process like this is that its utility is overstated as it assumes that cultural groups are a homogenous entity, when in fact in most countries there is extensive heterogeneity subsumed within a single setting. One solution to this involves developing the intervention to meet the needs of a smaller cultural sub-group within the larger culture, but this serves a narrow agenda [28]. An alternative would be to develop an intervention with decision rules which can be used to tailor its delivery depending on the needs of particular cultural subgroups [28]. While this would closely mirror individualised clinical practice, the clinical flexibility that would be required of such an intervention might be beyond the abilities of NSWs. Just as the treatment development process had limitations it also had several strengths. The biggest strength is the systematic and well-documented steps informed by robust scientific principles and peer reviewed sources e.g., MRC framework, and treatment development steps used by other published research. Besides this over-arching strength, there are several other strengths within the various steps of the treatment development process such as the detailed review of the existing evidence base and the contribution of various stakeholders to the participatory research processes.

The PM + intervention has been extensively adapted to make it suitable for scale-up [29]; and to respond to the unique requirements of target populations such as young people living with HIV in Kenya, refugee youth from Syria, Eritrea and Afghanistan, and women affected by gender-based violence and urban adversity in Kenya [30–32]. However, the CHANGE intervention is the first example of a PM + informed intervention which responds to the needs of conflict-affected populations with co-existing AUD and psychological distress. To conclude, our intervention development process paid close attention to the context, and such a systematic process is expected to enhance acceptability and feasibility, and eventually the effectiveness of a new intervention. After evaluation of acceptability and feasibility, a definitive evaluation of the effectiveness and cost-effectiveness of the final CHANGE intervention will be conducted through randomized controlled trials in Uganda and Ukraine. If demonstrated to be cost-effective, the CHANGE intervention would be an important tool to reduce the treatment gap for AUD in conflict settings using low-cost and scalable strategies.

## Supplementary Information

Supplementary Material 1.

## Data Availability

The datasets used and/or analysed during the current study are available from the corresponding author on reasonable request.

## Abbreviations

AUD: Alcohol use disorders
BI: Brief interventions
CBT: Cognitive behaviour therapy
CHANGE: AlCohol use in HumanitariAN settings: a programme of work to address alcohol use disorders and associated adversities among conflict-affected populations in UGanda and UkrainE
CRA: Community reinforcement approach
IDI: In-depth interviews
MHPSS: Mental health and psychosocial support
MI: Motivational interviewing
MRC: Medical research council
NSW: Non-specialist workers
PM+: Problem management plus
PTSD: Post traumatic stress disorde
TSP: 12-step treatment programme
