# Supplementary material for: Development of a non-specialist worker delivered psychological intervention to address alcohol use disorders and psychological distress among conflict-affected populations in Uganda and Ukraine
Source: Int J Ment Health Syst. 2025 Jan 8;19:2. doi: 10.1186/s13033-024-00656-4 (PMC11708126; doi:10.1186/s13033-024-00656-4)
Supplement: Supplementary file 1 — Supplementary Material 1. [file 13033_2024_656_MOESM1_ESM.docx]

## Appendix A: Topic guides for interviews

## Interview Guide: conflict-affected men who misuse alcohol

**Theme: Getting to know the participant**

- *Can you tell me a little bit about yourself?*

Probe: Number of family members, main provider for family, work/informal employment

- (Only if IDP, but not veteran) *Can you tell us more about life in* [name of settlement]?

Probe: How long, any difficulties experienced after displacement

- *Can you tell me about your health?*

Probe: Physical health, mental health problems

**Perceptions of problems with alcohol (descriptions of the problem, causes, and risk factors; and associated problems/e.g. violence)**

- *Please tell me about your alcohol consumption*

Probe: what, how much, where, when, with whom, how often

- *Can you tell me why you started drinking alcohol in the first place?*

Probe: If not able to articulate the reasons for starting to drink then probe for social events [easy accessibility]; peer pressure; to overcome adversities; to feel high; to get distracted from life; enjoyment

- *Under what circumstances do you tend to drink more than usual?*

Probe: if unable to articulate then probe for having a hard day, stress reliever, friends who drink, enjoyment, social events/occasions, sleep, physical pain reliver.

- *Under what circumstances do you tend to drink less than usual?*

Probe: Please give us some examples.

- *What kind of effects does alcohol have on you?*

Probe: Physical health, mental health, relationships, work, violence. Ask about perceived benefits as well as adverse effects. *I am interested both in positive and negative effects.*

**Theme: Help seeking behaviors, self-help and coping strategies used**

- *Have you ever tried to change your drinking?*

Probe: Did this strategy work? Ask about each strategy named by participant.

- Have you ever sought help from others to stop or control your drinking? *Can you tell us what type of help?*

Probe: type of services, formal health care providers, and non-formal providers, family, and friends

- Explore sequence of seeking help, what they found helpful and not helpful about each type of help sought.
- What has made it easier for you to look for help with stopping or controlling your drinking?
- *What has made it harder for you to look for help with changing your drinking*?

## Interview Guide: Family members

**Getting to know participant.**

- *Can you tell me a little bit about yourself?*

Probe: Number of family members, main provider for family, work/informal employment

- *Can you tell us more about life in* [name of settlement]?

Probe: How long, any difficulties experienced

- *Please tell me about health of your family member (who has problems with alcohol consumption)*

Probe: Physical Health, mental Health

**Perceptions of problems with alcohol (descriptions of the problem, causes, and risk factors and associated problems/e.g. violence)**

- *Under what circumstances does your family member tend to drink more than usual?*

Probe: if unable to articulate then probe for having a hard day, stress reliever, friends who drink, enjoyment, social events/occasions, sleep, physical pain reliver

- *Under what circumstances does your family member tend to drink less than usual?*
- *How does your family member’s drinking alcohol affect you?*

Probe: Physical health, mental health, relationships, work, violence.

- *How does your family member's behavior change when he does not consume alcohol?*
- *What do people living in your community think about alcohol consumption among veterans, military personnel, and their family members?*

Probe: Culture, traditions, beliefs, group norms.

**Theme: Help seeking behaviors, self-help and coping strategies used**

- *What services are available in your community for those who have problems with alcohol consumption?*
- *Has your family member ever sought help from others to change their alcohol-related behavior?* *If so, what kind of help did he seek to control his alcohol consumption?*

Probe: Official healthcare service providers, informal service providers, family, and friends.

- *Have you ever sought help from others to change your family member’s drinking? If yes, describe your experience of seeking for help?*

Probe: *What was helpful and not in each type of support?*

- *What has made it harder for you to look for help with changing your family member’s drinking*?

Interview Guide: Providers

**Theme: Mental health problems of people in the community**

- I would like to ask you about the kind of **mental health and psychosocial problems** people in your community may have.
  - *Can you tell me a little bit more about yourself and the work that you do?*
  - *What are the mental health and psychosocial people face in this community? Can you describe these problems?*

- *What do you feel are the three most important mental health and psychosocial concerns in this community?*

- *In your opinion what are the reasons for people developing these mental health and psychosocial problems? What causes these problems?*

- *Can you tell me more about* ***problems with alcohol*** *in this community? Can you tell me about the reasons for these problems?*

- Probe (for example): Traditions that influence alcohol use, group norms around alcohol use

**Theme: Current health seeking behaviors of persons with mental health and alcohol problems in the community**

- *In this community, where do people with* ***mental health problems*** *usually seek help?*

Probe: Probe about both, informal (e.g. family and friends, church, shrines) and formal sources of support (e.g., clinics)

- *What factors make it easier for people to seek help for mental health and psychosocial problems?*

- *What factors make it harder to seek help for mental health and psychosocial problems?*

- *In this community, where do people with* ***problems with alcohol*** *usually seek help?* (skip, if alcohol was listed before)

Probe: Probe about both, informal (e.g. family and friends, church, shrines) and formal sources of support (e.g., clinics)

- *What factors make it easier for people with alcohol problems to seek help?* (skip, if alcohol was listed before)

- *What factors make it harder with alcohol problems to seek help?* (skip, if alcohol was listed before)

- *What kind of help do the people with mental health and psychosocial problems coming to you ask for?*

- *What kind of help do the people who have* ***problems with alcohol*** *coming to you ask for?*

**Theme: MHPSS strategies/alcohol techniques delivered in the community and their perceived impact (PLEASE NOTE: ensure that participants describe what they actually do rather than just name the strategy itself such as motivational interviewing)**

- *What* ***mental health and psychosocial support services*** *are currently available in this community*?

- *What health services are currently available for* ***people who have problems with alcohol*** *in this community?*

- *What service do you provide for the priority mental health and psychosocial problems you have identified earlier?*

- *What has been your experience in working with people who experience mental health and psychosocial problems?*

Probe, if necessary: What makes it difficult/easy? What helps?

- *What service do you provide to people who have problems with alcohol*? (skip, if alcohol was listed before)

- *What has been your experience in working with people who have problems with alcohol?* (skip, if alcohol was listed before)

Probe: What makes it difficult/easy? What helps?
